# The SET and ankyrin domains of the secreted *Legionella pneumophila* histone methyltransferase work together to modify host chromatin

**DOI:** 10.1128/mbio.01655-23

**Published:** 2023-10-05

**Authors:** Monica Rolando, Ivy Yeuk Wah Chung, Caishuang Xu, Laura Gomez-Valero, Patrick England, Miroslaw Cygler, Carmen Buchrieser

**Affiliations:** 1 Institut Pasteur, Université de Paris, Biologie des Bactéries Intracellulaires, Paris, France; 2 Department of Biochemistry, Microbiology and Immunology, University of Saskatchewan, Saskatoon, Saskatchewan, Canada; 3 Institut Pasteur, Université de Paris, Plateforme de Biophysique Moléculaire, Paris, France; Weill Cornell Medicine, New York, New York, USA

**Keywords:** *Legionella pneumophila*, host-pathogen interactions, histone methyltransferase, epigenetics, H3K14

## Abstract

*Legionella pneumophila* is a bacterial pathogen ubiquitous in natural and man-made aquatic environments, where it replicates in protozoa. Its intracellular life cycle depends on the establishment of a *Legionella*-containing vacuole (LCV) where the bacteria replicate. To form the LCV, *L. pneumophila* depends on a type-4 secretion system that secretes more than 300 proteins in the host cell. Among these is RomA that encodes an N-terminal catalytic methyltransferase domain with a SET fold and a C-terminal domain containing six ankyrin repeats. We have reported that the SET domain has histone methyltransferase activity and specifically methylates lysine 14 of histone H3 during infection; however, the role of the ankyrin repeats is unknown. Here, we report the structure of RomA co-crystallized with a 15-mer peptide corresponding to the N-terminal tail of histone H3 at 2.2 Å. This structure revealed that the N-terminal arm of the peptide from Thr3-Ala7 binds to the ankyrin repeat domain and the C-terminal arm from Gly13-Arg17 and interacts with the SET domain, inserting Lys14 into the active site for methylation. Importantly, the interactions of the five N-terminal residues of the H3-peptide with the ankyrin domains account for ~50% of the peptide-RomA contacts, predicting that the ankyrin domains of RomA are essential for histone H3 binding and subsequent lysine methylation. *In vitro* enzymatic activity and binding assays confirmed these predictions. Thus, the six ankyrin domains are essential for chromatin binding, thereby allowing the SET domain to fulfill its histone transferase activity and to modify the host chromatin to the advantage of the pathogen.

**IMPORTANCE:**

*Legionella pneumophila* is an intracellular bacterium responsible of Legionnaires’ disease, a severe pneumonia that is often fatal when not treated promptly. The pathogen’s ability to efficiently colonize the host resides in its ability to replicate intracellularly. Essential for intracellular replication is translocation of many different protein effectors *via* a specialized secretion system. One of them, called RomA, binds and directly modifies the host chromatin at a unique site (tri-methylation of lysine 14 of histone H3 [H3K14me]). However, the molecular mechanisms of binding are not known. Here, we resolve this question through structural characterization of RomA together with the H3 peptide. We specifically reveal an active role of the ankyrin repeats located in its C-terminal in the interaction with the histone H3 tail. Indeed, without the ankyrin domains, RomA loses its ability to act as histone methyltransferase. These results discover the molecular mechanisms by which a bacterial histone methyltransferase that is conserved in *L. pneumophila* strains acts to modify chromatin.

## INTRODUCTION

*Legionella pneumophila* is a Gram-negative intracellular pathogen ubiquitous in natural environments and man-made water systems, where it replicates in numerous protozoa (1). Its intracellular life cycle has been very well characterized, especially the establishment of a *Legionella*-containing vacuole (LCV) through the remodeling of host vesicles and organelles, where the bacteria can safely replicate (2). When accidentally inhaled by humans *via* contaminated aerosols, *L. pneumophila* can replicate in lung macrophages and lung epithelial cells using the same mechanism as in protozoa thereby causing a severe form of pneumonia, called Legionnaires’ disease (3). The ability of *L. pneumophila* to establish the LCV depends on an efficient type-4 secretion system (T4SS), called Dot/Icm that is highly conserved throughout the genus *Legionella. L. pneumophila* secretes more than 300 effector proteins *via* this T4SS into the host cells that together play a concerted role in the establishment of the LCV, hence in the virulence of the bacterium (4).

Genome sequencing and analyses revealed that *Legionella* species encode proteins containing many different eukaryotic domains that have likely been acquired by horizontal gene transfer during coevolution with their protozoan hosts (5). Most interestingly, most of these so-called eukaryotic-like proteins are T4SS-secreted effectors and play an important role in the adaptation of *Legionella* to intracellular replication (6). The most abundant eukaryotic domains identified in the genus *Legionella* are ankyrin repeats, a motif of 33 amino acid residues well known for being involved in protein-protein interactions and participating in cellular signaling (7). In the *Legionella* genomes, they were found frequently associated with other eukaryotic motifs, hence constituting modular proteins (5). One example is the secreted effector RomA, encoded by the gene *lpp1683* in *L. pneumophila* strain Paris but present in all *L. pneumophila* genomes sequenced to date. This T4SS-secreted effector contains an N-terminal SET domain, responsible for the methylation of host histones (8) and several ankyrin repeats, located in the C-terminal moiety of the protein, for which the function is not known yet.

In eukaryotes, proteins containing SET domains encode lysine methyltransferase activity and were identified as targeting histone proteins. The first enzyme identified is the mammalian homolog of *Drosophila* SU-(var)3-9, which specifically targets Lys9 of histone H3 to mediate gene silencing (9). The catalytic motif was mapped to an evolutionary conserved, ~130-residue SET domain, named after three Drosophila genes, involved in epigenetic processes, *Su(var)*, *E(z)*, and *Trithorax* (10). Since then, SET domain-containing proteins have been shown to methylate lysine residues of a variety of histone and non-histone targets (11, 12). In 2002, structures of human SET7/9, *Neurospora* DIM-5, and *Schizosaccharomyces* Clr4 were published, providing preliminary insights into the mode of catalysis and substrate binding (13 – 15). SET domains often coexist with post-SET, SET-I, and pre-SET, and together, they contribute to cofactor (SAM) and substrate binding or to the structural stability of the protein. The insert region (SET-I) can have considerable variability in length and seems to play a role in substrate specificity (16). Specific residues conserved among the SET domain proteins play a catalytic role, particularly a tyrosine located in the C-terminal part of the domain functions as a general base to deprotonate the lysine substrate before the methyl transfer (17).

We have shown that the *L. pneumophila* effector RomA encodes a typical SET domain, where the N-terminal and C-terminal parts are highly conserved, including the GxG motif involved in SAM binding and the tyrosine involved in the active site (8). Furthermore, a unique target of RomA has been previously identified: tri-methylation of lysine 14 of histone H3 (H3K14) as revealed by mass spectrometry on H3 peptides and the full-length protein, following the *in vitro* incubation of RomA with purified histone H3, but also in THP-1 and *Acanthamoeba castellanii* cells infected by *L. pneumophila* (8). Intriguingly, it has been published that the homolog of RomA in *L. pneumophila* strain Philadelphia (named also LegAS4 encoded by *lpg1718*) targets histone H3 at lysine 4 (H3K4) *in vitro*; however, no specificity *in vivo* has been described (18). Furthermore, a crystal structure of the RomA homolog in strain Philadelphia has been published in 2015, revealing that the SET domain does show not only sequence similarity but also structural similarity to eukaryotic SET domains (19). Son and colleagues suggested that this protein indeed should function as a trimethyl-lysine transferase but the structure did not allow to define whether K4 or K14 was the specific substrate targeted. Additionally, they found that the ankyrin repeats are physically linked to the active site of the SET domain, which suggested that these repeats could have a role in the functioning of the enzymatic activity of the SET domain on its target.

Here, we report the crystal structure of the RomA homolog of strain Philadelphia in complex with S-adenosyl homocysteine (SAH) and a 15-mer peptide corresponding to the N-terminal tail of histone H3. We observed that its active site (Tyr233) is in front of Lys14 of histone H3, confirming that the two homologous *L. pneumophila* RomA proteins of strains Paris and Philadelphia target H3K14. A *L. pneumophila* strain Philadelphia deleted for *lpg1718* showed no methylation on H3K14 during infection of THP1 cells, in contrast to the wild-type strain. Furthermore, we show that the ankyrin repeats present in the C-terminal moiety of the RomA homologs are essential for its enzymatic activity towards histones.

## RESULTS

### The SET domain proteins RomA (strain Paris) and LegAS4 (strain Philadelphia) methylate H3K14

**Fig 1:**
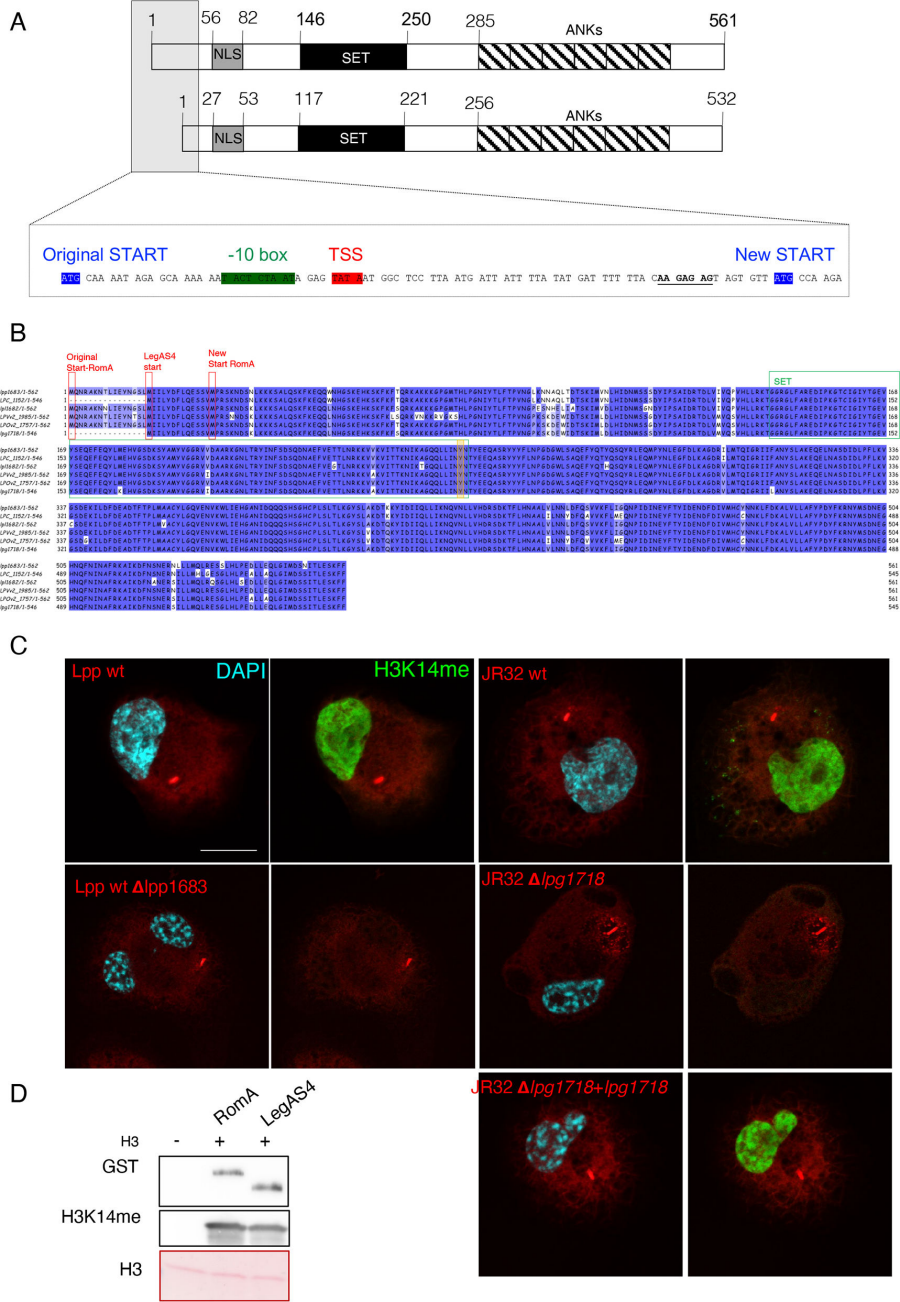
Sequence and functional comparison of the *L. pneumophila* effectors RomA and LegAS4. (**A**) Schematic representation of the RomA protein. NLS, nuclear localization signal; SET, SET domain; ANKs, ankyrin repeats. *Insert*: the predicted and new start sites identified by TSS mapping are depicted in blue. (**B**) Protein alignment of RomA homologs in different *L. pneumophila* strains (lpp, *L. pneumophila* strain Paris; LPC, *L. pneumophila* strain Corby; lpl, *L. pneumophila* strain Lens; LPVv2, *L. pneumophila* HL06041035; LPOv2, *L. pneumophila* strain Lorraine; lpg, *L. pneumophila* strain Philadelphia). Protein starts are highlighted in red; SET domain in green; active site Tyr in orange. (**C**) Immunofluorescence analysis of THP-1 cells infected 6 hours with wild-type *L. pneumophila* strain Paris (Lpp wt) and wild-type *L. pneumophila* strain Philadelphia (JR32 wt) as well with their derivatives, as indicated. JR32-Δ*lpg1718* strain was complemented with a plasmid encoding the gene *lpg1718* under control of its own promoter. Cells were stained for H3K14me2 (green), LPS (red), and DAPI (light blue). Scale bar 10 µm. (**D**) *In vitro* methyltransferase activity of RomA or LegAS4 against histone H3; 500 ng of GST-tagged enzymes in the presence of 500 ng of histone H3. The reaction was performed for 30 min at 30°C in the presence of cold SAM. GST-tagged proteins were detected with anti-GST antibodies; methylation of histone H3 by using specific H3K14me antibodies and H3 detected in the ponceau staining.

RomA was first identified and manually annotated in the frame of the sequencing project of *L. pneumophila* strain Paris. Gene *lpp1683* was predicted to encode a 561aa protein that carries a SET-domain and several ankyrin repeats (20). Subsequent analyses of the transcriptional profile of *L. pneumophila* strain Paris (21) identified a different transcriptional start site (TSS) for *lpp1683* and suggested that RomA is indeed shorter (532aa) than previously predicted, as the TSS was mapped to another start codon than predicted by annotation (Fig. 1A). The homolog of RomA in *L. pneumophila* strain Philadelphia, named LegAS4 (22) and encoded by the gene *lpg1718*, shares 96% homology with RomA. LegAS4 has been annotated as a 545aa-long protein, but this is still a prediction as a transcriptional start site mapping of strain Philadelphia has not been done yet. The 532aa versions of RomA and LegAS4 are 97% identical and contain both a nuclear localization signal (NLS) region (27–53aa), the functional SET domain (117–221aa), the conserved active site (Arg178, Asn181, and Tyr220), and a C-terminal domain-containing ankyrin repeats (256-532) (Fig. 1B). However, two different activities have been reported for these two proteins, which was puzzling given the high sequence similarity: for RomA, the specific methylation of H3K14 (8) and for LegAS4, the methylation of H3K4 (18). We previously tested by mass spectrometry analyses, performed on *in vitro* methylated histone H3, whether RomA could also methylate H3K4, but we did not identify any specific methylation of H3K4 (8). However, we additionally observed an increase in H3K14 methylation in cells infected with the *L. pneumophila* Philadelphia as we did for *L. pneumophila* Paris (23). To confirm that the H3K14 methylation induced during an infection with *L. pneumophila* strain Philadelphia is indeed conferred by its RomA homolog, we constructed a *lpg1718* deletion mutant and infected human macrophages (Fig. 1C). H3K14 methylation was detected in cells infected with wild-type *L. pneumophila* strains Paris and Philadelphia. In contrast, infections with the deletion mutants lacking *lpp1683* or *lpg1718*, respectively, did not lead to any methylation on Lys14. Complementation experiments confirmed that LegAS4 also specifically methylates H3K14 (Fig. 1C). *In vitro* histone methyltransferase activity (HMT) assays by using RomA or LegAS4 confirmed that the two effectors have the same enzymatic activity and both methylate H3K14 (Fig. 1D).

### Co-crystallization of LegAS4/RomA and the H3 peptide reveals the interaction interface

**Fig 2:**
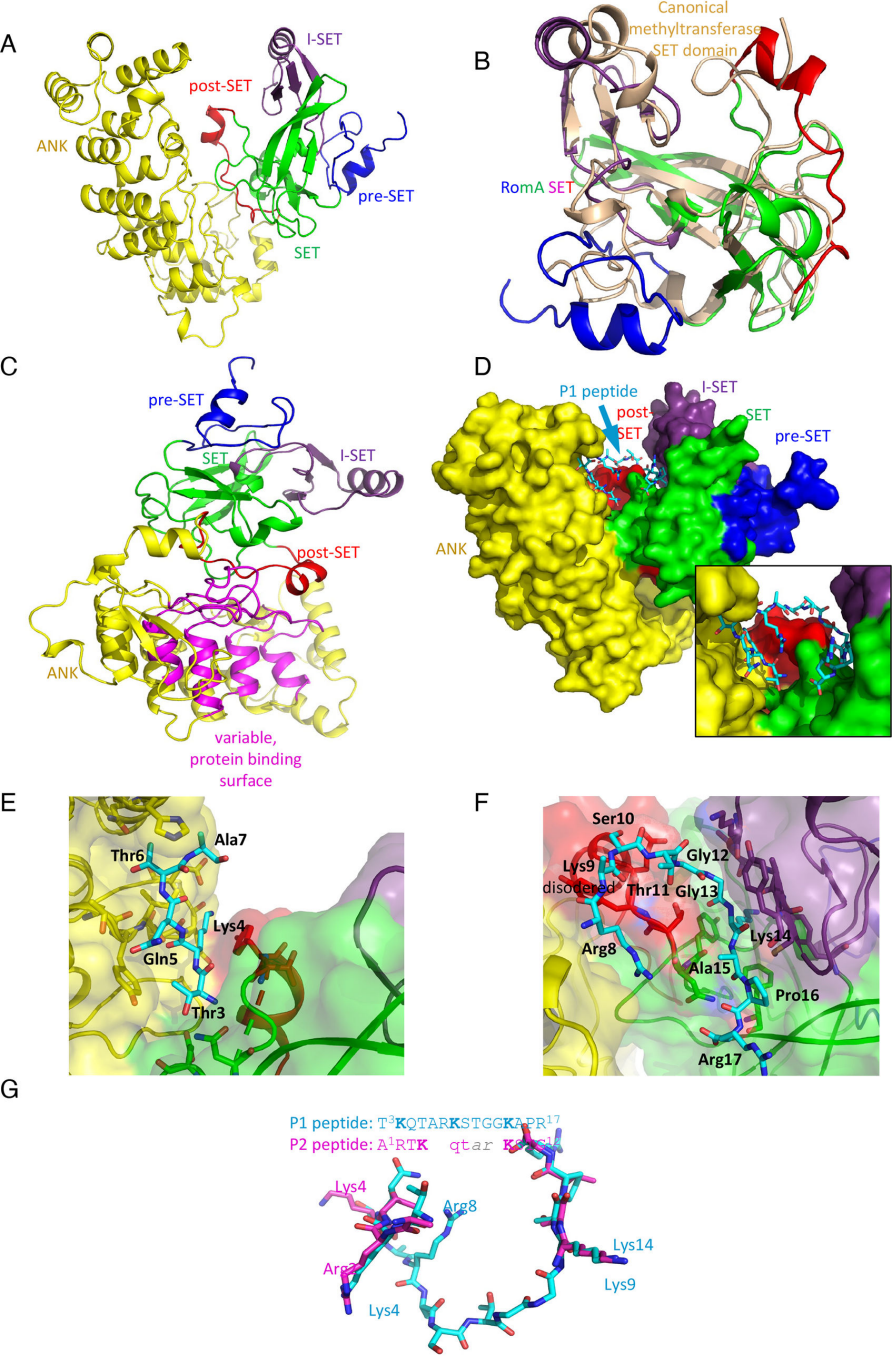
Crystal structure of LegAS4. (**A**) Cartoon representation of LegAS4. The segments are individually colored and marked. (**B**) Superposition of the RomA/LegS4 SET domain (rainbow color) with the canonical SET domain from histone methyltransferase SET7/9 (wheat color). (**C**) Orientation of LegAS4 showing the interaction between the SET domain (green) and post-SET segment (red) with the ANK domain and, in particular, the top of the loops forming variable protein binding surfaces (magenta). (**D**) The space filling model of LegAS4 with the P1 peptide bound. P1 is bound in the crevice between the SET and ANK domains making contacts with both. Insert shows a zoomed view on the peptide. (**E**) The interaction between the N-terminal segment of peptide P1 (blue) and the ANK domain (yellow). (**F**) The C-terminal segment of peptide P1 (blue) interacts with the post-SET domain (red) and the SET domain (green) and the I-SET segment (magenta). (**G**) Superposition of peptides P1 and P2. In the absence of Lys 14 in P2 peptide, Lys9 instead enters the active site. The N-terminal segment interacts with the same surface of ANK domain as P1; however, the shorter middle segment is disordered.

To gain insight into the function of the ankyrin and the SET domains within RomA/LegAS4, we co-crystalized LegAS4 with a 15-mer peptide corresponding to the N-terminal tail of Histone H3. The co-crystallization analyses was aimed at understanding how the effector recognizes Lys14 and at learning whether the ankyrin repeats located in the C-terminal part of the protein play a role in peptide binding. For this co-crystallization and the structural analyses of LegAS4 (84–532aa), the RomA homolog of *L. pneumophila* strain Philadelphia, we used two peptides: a 15-mer NH2-T_3_K_4_QTARK_9_STGGK_14_APR_17_-COOH (P1) that encompassed K14 and K4, as both have been described to be methylated by RomA or LegAS4, respectively, and a 12-mer NH2-A_1_RTK_4_QTARK_9_STG_12_-COOH peptide that contained K4 and K9, but not K14. The structure with the 15-mer peptide was resolved at a 2.22 Å resolution and the one with the 12-mer peptide at a 3.0 Å resolution. These structures reveal that the LegAS4 structure superimposes very well with apo LegAS4 (PDB ID 5CZY (16),) with a root-mean-square deviation (rmsd) of 0.56 Å for the Ca atoms and 1.03 Å for all atoms. These rmsd values are similar to those for the superposition of LegAS4 from the complexes with peptides P1 and P2, indicating that no significant conformational changes occur upon peptide binding. LegAS4 is composed of two domains, the N-terminal catalytic methyltransferase domain with a SET fold as described for typical human SET domain-encoding proteins (16) and a C-terminal domain with six ankyrin repeats, including the N- and C-capping structures (16) (Fig. 2A). The SET domain is similar to other human histone methyltransferases, such as GLP/EHMT1, G9a/EHMT2, PRDM2, and SUV39H2 (24), and is composed of four segments (16): the pre-SET segment (84–114aa), the core SET domain (115–147aa and 190–234aa), insertion I-SET segment (148–189aa), and post-SET segment (235–253aa) (Fig. 2B) (22) (16). The structure of LegAS4 that we obtained is similar to the one previously solved by Son and colleagues (19), which revealed that the SET domain and the ankyrin repeat domain are joined by a helix linker. These authors suggested that the extensive interactions with ankyrin repeats contribute to the stabilization of the SET domain. Indeed, our structure also indicates that the SET domain binds on the top of the ankyrin domain, contacting the long loops between the ankyrin repeats through the tips of two loop, 123–127aa and 197–199aa, and through the entire post-SET segment (Fig. 2C). The concave surface of the ankyrin domain, which is the most variable in ankyrin sequences and is usually associated with other proteins, is free to interact with other cellular targets (7). Moreover, there is a deep tunnel in the center of this surface with a slight hydrophobic character that could recognize a specific sidechain. We can speculate that, similarly to the ankyrin domain of GLP and G9a, this tunnel might accommodate a methylated lysine (25).

Analyzing how the peptide fits into the active site of the protein, we observed that the peptide P1 is well defined along the entire sequence (a representative electron density is shown in Fig. S1). Although it contains three lysine residues in the sequence that could potentially enter the active site cleft, with the SAH cofactor at the bottom, it is clearly Lys14 that enters the active site in the context of this peptide. The peptide assumes a U-shape conformation with the arms following grooves on either side of a bulge created by a short, two-turn α-helix at the beginning of the post-SET segment (235–253aa) (Fig. 2D). The N-terminal arm of the peptide, Thr3-Ala7, binds to the ankyrin repeat domain, and the C-terminal arm (Fig. 2D-insert), Gly13-Arg17, interacts with the SET domain, inserting Lys14 into the active site for methylation. The residues Lys9-Ser10-Thr11-Gly12 make a shallow turn. The Thr3 sidechain hydrogen bonds with Asn232 and weakly with Asp197 from the SET domain and extends the Cβ toward Tyr446 from the ankyrin domain. The Lys4 sidechain enters the groove between post-SET and ankyrin domains, H-bonds with Asp451 and the carbonyl of Asp448 (ankyrin domain), and stacks against Tyr236 (post-SET). In addition, its carbonyl group forms H-bonds with the NH of Ile447. The Gln5 sidechain establishes H-bonds with Thr445 and partially stacks against Tyr446. Thr6 enters a deep pocket that can accommodate a longer sidechain and forms weak H-bonds with Asn493 and the carbonyl of Thr445. Finally, Ala7 stacks against Phe492 (Fig. 2E; Table 1). The Arg8 sidechain extends along the backbone of Thr234-Tyr236 of the post-SET and forms H-bonds back to the sidechain of Gln5. The sidechain of Lys9 is disordered and makes no contacts with LegAS4, but its carbonyl makes contacts with Tyr236. Ser10 crosses over the post-SET chain while Thr11 bends toward Thr235 and the carbonyl of Tyr236. Gly12 and Gly13 continue the bend allowing Lys14 to enter the cleft leading toward partially buried SAM. Pro16 abuts a shallow depression in the SET domain surface, and finally, Arg17 extends along the Glu206 sidechain adding charge-charge interaction to the binding. Thus, all residues from peptide P1 contribute to binding (Fig. 2F; Table 1). Importantly, interactions of the five N-terminal residues with the ankyrin domain account for ~50% of the P1-LegAS4 contacts, suggesting that the ankyrin domain of LegAS4 is essential for the binding of the N-terminus of histone H3 and for subsequent lysine methylation.

**TABLE 1:** Contacts of the (3-17)-N-terminal H3 P1 peptide with ankyrin domain (ANK), post-SET, and core-SET^*a*^ ^*c*^

H3 peptide	ANK	Post-SET	Core-SET
Thr3		**Asn232**	Asp197
Lys	Asp409 **Ile447^*b*^** **Asp451** **Asp448 (back**) **Glu449 (back**)	**Tyr236 (stacking**)	
Gln5	**Thr445** Tyr446 (stacking)		
Thr6	**Thr445 (backbone**) **His489** **Asn493^*b*^**		
Ala7	**His489** Phe492		
Arg8			**Asn234** **Thr235**
Lys9 (backbone)		**Tyr236**	
Ser10		**Tyr236** **Glu237** **Glu238 (backbone**)	
Thr11		**Thr235**	
Gly12		**Thr235** **Glu237**	
Gly13		**Thr235**	**Lys170 (backbone**) **Ala173 (backbone**) Tyr233
Lys14			**Tyr148** **Ser171 (backbone**) **Ala173 (backbone**) **Met174** **Tyr175 (backbone**) **Tyr233** **SAM**
Ala15			**Met174** Phe204 Tyr233 (backbone)
Pro16 (backbone)			**Tyr175 (backbone**) **Val176** Phe204 **Asn234**
Arg17 (backbone)			**Phe204 (backbone**) **Asn234**
Arg17			**Glu206**

^*a*^ Core-SET: 115–234aa; post-SET: 235–253aa; ANK: 256–545aa. Active site: Tyr233, corresponding to Tyr220 in the 532aa form and Tyr249 in the 561aa form.

^*b*^ Key residues connected to H3 peptides.

^*c*^ Contacts shorter than 3.65 Å are shown in bold; remaining are contacts closer than 4 Å.

To test if the substrate binding site could potentially accommodate different conformations of the H3 N-terminus that would allow Lys4 or Lys9 to enter the active site, we have determined the structure of LegAS4 (84–532aa) with a peptide corresponding to the first 12 residues of histone H3 (peptide P2). This peptide is not fully visible in the electron density; only residues Ala1-Thr6 and Lys9-Gly12 could be modeled, with Lys9 in the active site. Overall, the P2 peptide follows the same path as peptide P1 forming a U shape, but the residues Ala7-Arg8 that are at the bottom of the U are disordered. Overall, the P2 peptide contacts the same regions of LegAS4 and interacts with both the ankyrin repeat domain with its N-terminus and the SET domain at its C-terminus (Fig. 2G). Ala1 forms H-bonds through its amino group with the Asp97 to Asp197 sidechain, and the Ca makes van der Waals contact with the sidechain of Phe195 (SET domain). Arg2 forms a salt bridge to Asp451 (ankyrin domain) and its carbonyl H-bonds to NH of Ile447. Thr3 forms H-bonds to Thr445 (ankyrin domain). Lys4 sidechain H-bonds to Asn493 (ankyrin), and its NH H-bonds with carbonyl of Thr445. Gln5 forms a weak H-bond with His489, and carbonyl of Thr6 weakly H-bonds to NH of Tyr236 (post-SET). The next visible residue, Lys, enters the active site extending toward the SAH cofactor. Its carbonyl weakly H-bonds to NH of Tyr175 (I-SET). Ser10 and Thr11 follow the groove making van der Waals contacts with neighboring residues from the SET and I-set segments. Finally, Gly12 makes a weak H-bond from its NH to the carbonyl of Phe204 (SET domain).

The observation that both peptides utilize the same two patches on the LegAS4 surface, one in the ankyrin domain and the other on the SET domain, strongly suggests that both patches contribute to an extended binding site, with the ankyrin site adding significantly to substrate selectivity. The superposition on the two peptides, P1 and P2, based on their structures bound to LegAS4 shows the preference for residues in each position (Fig. 2G). However, in the context of the entire histone H3, our structures suggest that it is Lys14 that is predominantly embedded into the active site, making it the prime methylation target of LegAS4. The structure with peptide P2 indicates that Lys9 can also enter the active site. However, this conformation must have a significantly lower affinity than that with Lys14 in the active site; because, in the structure with peptide P1, we can only model conformation with Lys14 in the active site, we hypothesize that Lys9 could also be methylated but only at a basal level.

### The ankyrin domains are essential for the enzymatic activity of RomA/LegAS4

**Fig 3:**
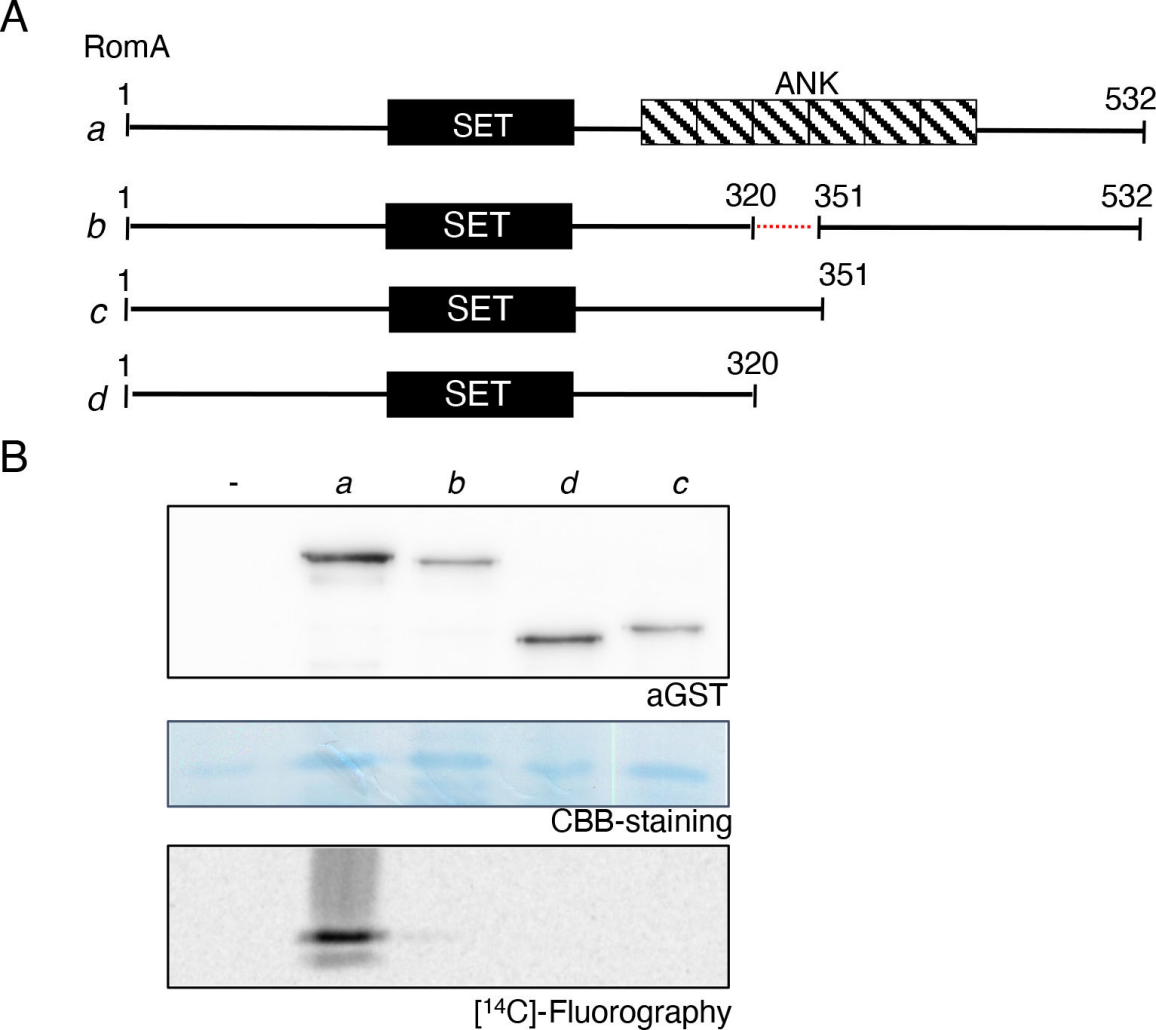
Ankyrin domains are essential for enzymatic activity of RomA. (**A**) Schematic representation of the RomA protein and the different deleted forms used in this study. (**B**) *In vitro* histone methyltransferase (HMTase) activity of RomA proteins using histone H3 as substrate. One microgram of recombinant GST-tagged proteins was incubated 1 hour at 30°C in the presence of 1 µg of histone H3 and 200 nCi of [^14^C]-SAM as substrate. GST-tagged proteins were detected with anti-GST antibodies and H3 loading with Coomassie Brillant Blue (CBB) staining.

The structure analyses suggested that the ankyrin domains are also essential for the methyltransferase activity of RomA/LegAS4 on histones. To analyze this prediction, we undertook mutational analyses of RomA followed by testing its *in vitro* histone methyltransferase activity (Fig. 3A). We tested the activity of different forms of RomA truncated in its C-terminal part by partially removing the ankyrin folds (Fig. 3B, constructs *c* and *d*) for their activity, compared with those of the full-length RomA. As presented in Fig. 3B, all deletions introduced in the C-terminal part of the enzyme abrogated methyltransferase activity of RomA against H3 proteins. Furthermore, to not to disturb the overall structure of the protein too much, we also tested an enzyme deleted for only one ankyrin repeat in the center of the domain (31aa, construct *b*). Again, we did not detect any methyltransferase activity on histone H3. This showed that when the C-terminal part of the enzyme is partly or completely disturbed, RomA loses its enzymatic activity against H3, confirming the essential role of the C-terminal located ankyrin repeats for its function.

**Fig 4:**
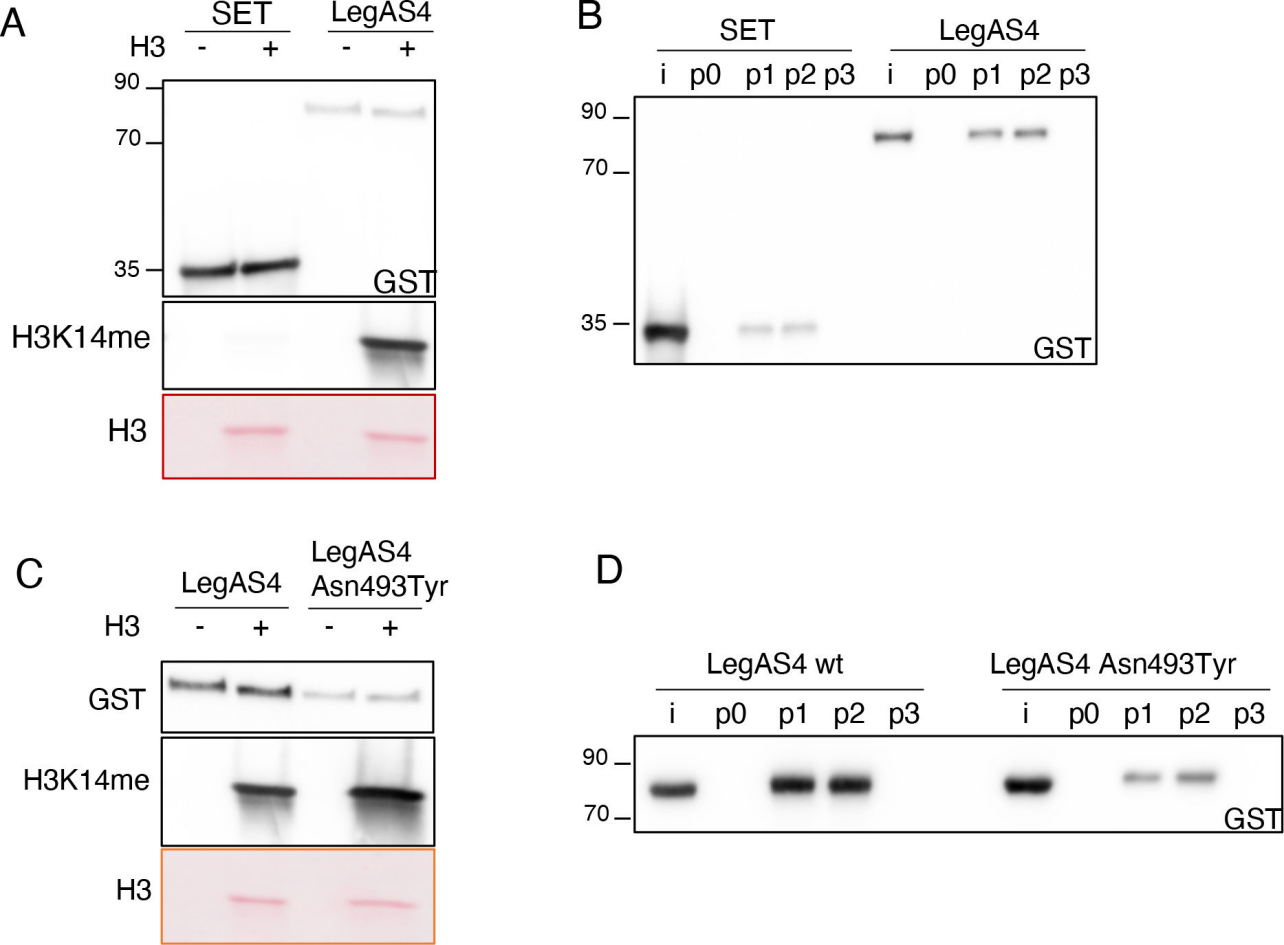
Role of ankyrin repeats on LegAS4 activity. (**A**) Comparison of the methyltransferase activity of the SET domain or full-length LegAS4 against histone H3 at Lys14. *In vitro* HMT assay of 500 ng of GST-tagged enzymes in the presence of 500 ng of histone H3. The reaction was performed for 30 min at 30°C in the presence of cold SAM. GST-tagged proteins were detected with anti-GST antibodies, methylation of histone H3 by using specific H3K14me antibodies and H3 detected in the ponceau staining. (**B**) *In vitro* binding of the SET domain or full-length LegAS4 to H3_1-21aa_ (**P1**) ,H3_1-21aa_Thr6Tyr (**P2**), and H3_1-7aa_ (**P3**). I, input; p0, no peptide. Biotin-conjugated histone peptides were immobilized on Streptavidin-coated beads and their binding to GST-tagged proteins detected by using specific anti-GST antibodies. (**C**) Comparison of methyltransferase activity of LegAS4 or LegAS4-Asn493Tyr against histone H3 at Lys14. *In vitro* HMT assay of 500 ng of GST-tagged enzymes in the presence of 500 ng of histone H3. The reaction was performed for 30 min at 30°C in the presence of cold SAM. GST-tagged proteins were detected with anti-GST antibodies, methylation of histone H3 by using specific H3K14me antibodies, and H3 detected in the ponceau staining. (**D**) *In vitro* binding of LegAS4 or LegAS4-Asn493Tyr to H3_1-21aa_ (**P1**), H3_1-21aa_Thr6Tyr (**P2**), and H3_1-7aa_ (**P3**). I, input; p0, no peptide. Biotin-conjugated histone peptides were immobilized on Streptavidin-coated beads and their binding to GST-tagged proteins detected by using specific anti-GST antibodies.

To further evaluate the role of the ankyrin domains in peptide binding and consequently in the methyltransferase activity of RomA/LegAS4, we performed peptide binding assays and *in vitro* HMT assays using purified proteins. These experiments showed again that the SET domain alone is not able to methylate H3 proteins (Fig. 4A). We then assessed the ability of the wild-type LegAS4 protein to bind different H3 peptides. Structural analyses showed that Thr6 of H3 can form bonds with Asn493, located in the ankyrin domain of LegAS4 (Table 1). Thus, we first mutated Thr6 (Thr6Tyr) on the H3 peptide; however, this single-point mutation did not affect the binding to the H3 peptide suggesting that Thr6 is not the only residue on the H3 peptide responsible for binding the enzyme in its C-terminal part (Fig. 4B). However, as we observed that a shorter form of the H3 peptide (Ala1-Ala7) was not binding the enzyme, this suggests that Thr6 and the region surrounding play an important role in the binding to Lys 14, further arguing against a methylation of Lys4 by LegAS4 (Fig. 4B). We then mutated Asn493 to Tyr, produced this mutated form of LegAS4 in *E. coli*, and undertook *in vitro* HMT assays to assess the role of a Asn493Tyr point mutation in binding H3 peptides. Whereas LegAS4-Asn493Tyr was still able to methylate the H3 proteins *in vitro* (Fig. 4C), its binding to the H3 peptides (either wild-type H3_1-21aa_ or Thr6Tyr) was strongly affected (Fig. 4D). Thus, Asn493 is a key residue for H3 peptide binding.

**Fig 5:**
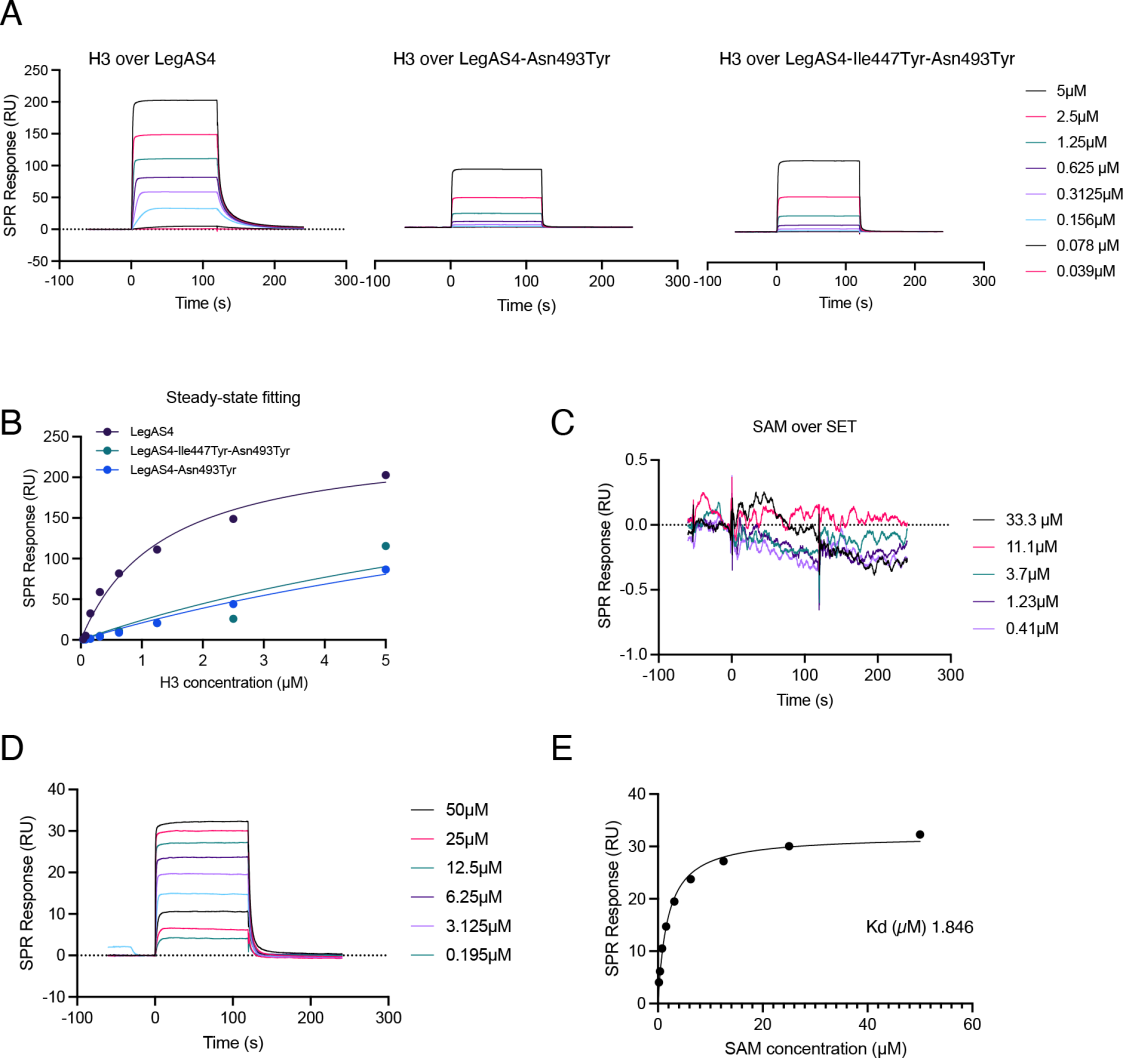
Binding of the H3 peptide and SAM to immobilized LegAS4 proteins assessed by SPR. (**A**) Peptide injections over immobilized LegAS4 or LegAS4-Ile447Tyr-Asn493Tyr or LegAS4-Asn493Tyr. Reference and blank traces were subtracted for ease of viewing. Injection concentrations of H3 peptides (residues 1–21), as indicated. The association/injection is from 0 to 120 s and the dissociation/buffer flow is from 120 to 240 s. (**B**) Steady-state analysis of H3 peptide binding over LegAS4 enzymes. Dissociation equilibrium constants (*K*_d_s) were determined by fitting the concentration dependence of steady-state SPR responses. LegAS4: *K*_d_ 1.38 ± 0.08 µM; LegAS4-Ile447Tyr-Asn493Tyr: *K*_d_ = 9.15 ± 0.19 µM; LegAS4-Asn493Tyr: *K*_d_ = 8.73 ± 0.09 µM; H34me: *K*_d_ = 7.8 ± 0.2 µM. (**C and D**) SAM injections over the immobilized (C) SET domain and (D) full-length LegAS4 protein. Reference and blank traces were subtracted for ease of viewing. Injection concentrations of SAM, as indicated. The association/injection is from 0 to 120 s, and the dissociation/buffer flow is from 120 to 240 s. (E) Steady-state analysis of SAM binding over full-length LegAS4 protein. Dissociation equilibrium constant (*K*_d_ = 1.846 µM) was determined by fitting the concentration dependence of steady-state SPR responses.

To get insight into the contribution of the various contacts between LegAS4 and the H3 peptide, we performed detailed binding analyses using surface plasmon resonance (SPR) assays, a method that allows quantitative real-time analysis of interactions. We covalently immobilized different forms of LegAS4 onto sensor chips: wild type, Asn493Tyr, and a Ile447Tyr-Asn493Tyr, with an additional mutation of a key residue involved in Ank-H3 binding based on the structure (Table 1). The change in response level was proportional to the change in mass at the surface when the interacting partner in solution (the analyte) binds upon injection over the ligand-functionalized surface. Increasing concentrations of the H3_1-21aa_ peptides were brought in contact with immobilized wild-type LegAS4 through the flow cells of the SPR instrument, and the steady-state analyses yielded a *K*_d_ value of 1.38 ± 0.08 µM (Table 2). The same experiment performed with LegAS4-Asn493Tyr and LegAS4-Ile447Tyr-Asn493Tyr confirmed the role of the residues deemed crucial for peptide binding according to the structure (Fig. 5A and B; Table 2). Consistent with the importance of the interactions between the ankyrin domains and the H3 peptide, the mutation of Asn493 to tyrosine caused a significant decrease in peptide binding constant, compared with the wild-type enzyme.

**TABLE 2:** Binding kinetics of the H3 peptide and SAM to different LegAS4 forms, estimated by SPR-based real-time binding assays

Product	H3-peptide binding (SPR) (µM)	SAM binding (SPR) (µM)
LegAS4	1.38 ± 0.08	2.00 ± 0.15
LegAS4-Asn493Tyr	9.15 ± 0.19	1.94 ± 0.13
LegAS4-AIle447Tyr-Asn493Tyr	8.73 ± 0.09	2.07 ± 0.16

Given that the SET domain alone still binds the H3 peptides, although at very low level, whereas no activity at all was detected in *in vitro* HMT assays performed on H3 proteins (Fig. 4A and B), we quantified its binding to the SAM donor. We performed SPR assays as previously described, by bringing the immobilized proteins in contact with increasing amounts of SAM. Interestingly, this showed that the different proteins had the same binding capacity to SAM (Table 2), but the SET domain alone showed greatly decreased SAM binding (Fig. 5C), compared with the full-length enzyme (Fig. 5D and E). This suggests that the presence of the ankyrin domains is crucial to form the correct conformation of the SAM binding site in front of the catalytic site of the SET domain.

## DISCUSSION

“Epigenetics and infection” is an emerging field of research (26 – 28). Indeed, more and more bacterial effectors that target host chromatin have been identified in the last decade (29), among them RomA, an effector that plays an important role in intracellular replication of *L. pneumophila (8*). All sequenced *L. pneumophila* strains encode a homolog of RomA, and seven different groups of orthologous proteins containing SET domains have been identified in the genus *Legionella* with some species having acquired more than one SET domain (30). Previously, we have shown that RomA uniquely targets H3K14 (8), a histone mark that was also confirmed in infection of host cells with *L. pneumophila* strain Paris as well as for seven different *L. pneumophila* strains tested including strain Philadelphia (23). However, a specificity for lysine 4 has been described for the RomA homolog in *L. pneumophila* strain Philadelphia named LegAS4. This observation led to speculations about the possibility that one bacterial effector may carry two distinct specificities, due to a 16-amino acid difference in the N-terminal part of the protein and an 8-amino acid stretch showing only weak sequence homology between the two proteins (31) (Fig. 1B). However, transcriptional start site mapping of strain Paris corrected the start codon proposed in 2004 (20) leading to a 32-amino acid shorter RomA protein (Fig. 1) (20). As TSS mapping has not been performed for strain Philadelphia, an exact comparison of the N-terminal part of the two homologous effectors and their TSS is not possible. Independently of the start of the protein, our mutational analyses of the RomA homolog in strain Philadelphia showed that it methylated H3K14 as RomA of strain Paris and that the mutant missing LegAS4 (*∆lpg1718*) loses methylation activity and its complementation restored H3K14 methylation. Thus, LegAS4 and RomA both target H3K14 (Fig. 1).

The analysis of different groups of orthologous proteins containing SET domains in the genus *Legionella* revealed that the SET domain present in *L. pneumophila* strains is always associated with an ankyrin domain (30). Ankyrin repeats play a role in protein-protein interactions and are often enriched in environmental intracellular bacteria and found in modular proteins in combination with several different eukaryotic-like domains (5, 32). In *Legionella*, ankyrin repeats were found in more than 300 putative effectors (33), and associations with functional domains such as domains that mediate ubiquitination or small GTPases were suggested to engage host proteins and/or confer specificity for target proteins. Interestingly, a first hypothesis regarding the function of ankyrin domains was that it directly interacts with nuclear host proteins, reminiscent of the direct interaction of methyltransferase G9a/GLP, *via* its ankyrin repeat domains, with DNMT3A/B (34), that is essential for the protection of imprinted DNA methylation (35). The observation that ankyrin domains of RomA are necessary for its methyltransferase activity on histone H3 *in vitro* suggested that these repeats could help in substrate binding more than in protein interaction (e.g., bacterial or host proteins), as it was observed for the effector AnkX, that binds the endogenous protein PLEKHN1 *via* a central fragment containing eight ankyrin repeats (36). Other examples of ankyrin-dependent engagement of host proteins are LagA15, a GTPase that targets host lipid droplets through its ankyrin repeats and leads to Golgi apparatus fragmentation (37), or AnkH, that binds endogenous LARP7 via the β-hairpin loop of its third ankyrin repeat (38).

By using RomA in *in vitro* HMT assays, we observed that a complete or partial deletion of the ankyrin repeats located in the C-terminal part of the protein abrogates the enzymatic activity of RomA on histone H3 (Fig. 2). Thus, we hypothesized that the function of the ankyrin repeats might be important for substrate binding, like the ankyrin repeats of the G9a/GLP methyltransferases that function as mono- and dimethyl-lysine binding modules (25). The analyses of our crystal structure confirmed the direct binding of ankyrin repeats to the H3 peptide and revealed that this *L. pneumophila* effector is organized in two distinct domains: the SET domain in the N-terminal part and the ankyrin repeats, located in the C-terminal part. These two moieties were previously reported to extensively interact, especially through the SAM-binding amino acids (19). Indeed, the co-crystal showed that these two domains squeeze on the H3 peptide and each domain has tight contacts with each other (Fig. 3). Importantly, the catalytic tyrosine is found in front of Lys14 of H3, together with the SAM (Table 1), confirming the preferred target of the enzyme. The essential role of ankyrin repeats in driving the activity of this *L. pneumophila* effector was confirmed when a C-terminal deletion of the protein showed no enzymatic activity, neither peptide binding (Fig. 4). The capacity of ankyrin repeats to bind the H3 peptide was also shown through binding analyses using surface plasmon resonance assays. Furthermore, single amino acid mutations on Ile 447 and Asn493 confirmed the direct binding of the ankyrins to the target peptide (Fig. 5).

Taken together, our structural analyses of the *L. pneumophila* LegAS4 effector containing a SET domain and six ankyrin domains and crystalized together with the H3 peptide showed that the ankyrin and the SET domains are essential for the enzymatic activity and that the amino acid Asn493 is an important residue for H3 peptide binding. Our phylogenetic and evolutionary analyses have shown that the SET domain has probably been acquired by horizontal gene transfer from a protozoan host of *L. pneumophila*. Here, we have undertaken a phylogenetic analysis of the ankyrin domain. As shown in Fig. S2 similarly to the SET domain, the ankyrin domain seems also to have been acquired by horizontal gene transfer. However, how this modular protein has been “assembled” by *L. pneumophila* to work in concert to methylate host histones remains an open question.

## MATERIALS AND METHODS

### Bacterial strains, culture conditions, and DNA manipulations

*L. pneumophila* strain Paris (Lpp) and strain Philadelphia (JR32) as well as their derivatives were cultured in N-(2-acetamido)−2aminoethanesulfonic acid (ACES)-buffered yeast extract broth or on ACES-buffered charcoal-yeast (BCYE) extract agar (39). For cloning, *Escherichia coli* strain DH5α (Invitrogen) was grown in Luria-Bertani (LB) broth and agar. For knockout and complementation constructions, antibiotics were added at the following concentrations: ampicillin at 100 µg/mL and kanamycin at 50 µg/mL for *E. coli*, chloramphenicol at 20 µg/mL for *E. coli* and 10 µg/mL for *L. pneumophila*, and gentamycin at 50 µg/mL for *E. coli* and 12.5 µg/mL for *L. pneumophila*. All strains were grown at 37°C. DNA manipulations and restriction enzyme digestions were performed using standard procedures. To generate truncated forms of RomA (Figure 3, constructs *c* and *d*), the corresponding truncated gene was PCR cloned with *BamH*I-*Xho*I sites into pGEX-4T2: construct *c*, *fwd*-primer: ggatccatgcaaaatagagcaaaaa, *rev*-primer: ctcgagtcattgttgttgatcgatattagcgc; construct *d*, *fwd*-primer: ggatccatgcaaaatagagcaaaaa, *rev*-primer: ctcgagtcacgcctcatcaaaatctaatattttttc; SET domain *fwd*-primer: ggatccgggcgaggtttatttgc, *rev*-primer: ctcgagtcaattatagttgattaatagttgttgtcctg. To generate the construct deleted for one ankyrin repeat (construct *b*), specific primers that hybridize to regions on either side of the area to be deleted were designed. The primers contain *SacI*I-ends, allowing the two ends to be ligated together following inverse PCR amplification and *Dpn*I digestion (*fwd*-primer: aaaccgcggagtcattctggtcattgtccttt; *rev*-primer: aaaccgcggcgcctcatcaaaatctaatatttttt). DNA sequencing was used to confirm the deleted constructs. The *∆lpg1718* mutant was constructed as described previously (40) and complemented with the full-length *lpg1718* gene with an N-terminal myc-tag under the control of its own promoter cloned into pBC-KS (Stratagene). Plasmids expressing the glutathione S-transferase (GST) protein fusions were constructed starting from pGEX vectors (Stratagene). *romA* (*lpp1683*) was cloned into pGEX4T2 (GE-Healthcare) as detailed in (8).

### Cloning constructs for crystallization, protein expression, and purification

Residues 84–532 of *legAS4* from *L. pneumophila* strain Philadelphia (Uniprot: Q5ZUS4) were cloned into vector pRL652, a derivative of vector pGEX-4T-1 (GE Healthcare) adapted for ligation-independent cloning. The plasmid was transformed into BL21(DE3) (Agilent) for protein expression. The expressed protein contains a TEV-cleavable GST tag at the N-terminus. For large-scale expression, a 20-mL overnight culture in LB supplemented with 100 µg/mL of ampicillin and 0.4% glucose were inoculated into each liter of Terrific Broth media with a total volume of 6 L. All media used were supplemented with 100 µg/mL of ampicillin. The inoculated cultures were grown at 37°C until they reached OD_600_ of 1.0 and were then induced with 0.5 mM of isopropyl β-D-1-thiogalactopyranoside (IPTG). The cultures were induced at 20°C and incubated overnight. The cells were harvested by centrifugation at 9,110 × *g* for 15 min. The cell pellet was resuspended in 50 mL of PBS supplemented with 2 mM benzamidine. The re-suspended cell pellet was lysed using a cell disruptor (Constant Systems Ltd). The cell debris was removed by centrifugation at 28,964 × *g* for 30 min. The clarified lysate was applied to 15 mL of Glutathione-Superflow resin (Clontech) equilibrated with lysis buffer. The binding was performed at 4°C for 1 hour followed by a 100 mL lysis buffer wash. The beads were resuspended in 20 mL of lysis buffer and incubated with TEV protease (33 µg/mL) overnight at 4°C. After TEV cleavage, the flowthrough containing the cleaved LegAS4 was dialyzed against 1 L of buffer containing 20 mM Tris pH 8.0 and 50 mM NaCl overnight at 4°C. The dialyzed sample was loaded on a MonoQ anionic exchanger column (GE Healthcare) and equilibrated with Buffer A (20 mM Tris pH 8.0). The protein was eluted with a continuous gradient of Buffer B (20 mM Tris pH 8.0 and 1.0 M NaCl) at 0%–50% over 20 column volumes. The fractions with LegAS4 were pooled and further purified by gel filtration on a SEC650 (Bio-Rad) column eluted with solution containing 20 mM Tris pH 8.0 and 100 mM NaCl. For crystallization, the sample was concentrated to 30 mg/mL using Amicon centrifugal filter (Millipore) with a molecular weight cut-off of 10,000 Da. The concentration was measured with Nanodrop UV Spectrophotometer (Themo Scientific) using an extinction coefficient of 55,490, calculated by ProtParam (41).

### Crystallization and data collection

The protein was co-crystallized with histone H3 peptide P1 (NH2-T_3_K_4_QTARK_9_STGGK_14_APR_17_-COOH) or peptide P2 (NH2-A_1_RTK_4_QTARK_9_STG_12_-COOH) (LifeTein, New Jersey, USA). The crystals were obtained at room temperature (~20°C) by the hanging-drop method. Two microliters of solution containing 30 mg/mL of protein, 1 mM histone H3 peptide dissolved in water, and 1 mM S-Adenosyl-L-homocysteine (Milipore Sigma, USA) was mixed with 1 µL of reservoir solution containing 0.1 M Bis-Tris pH 6.5 and 38% polypropylene glycol P400. For co-peptide 2, crystallization of the polypropylene glycol P400 concentration was decreased to 25%. The drop was suspended over the well containing 0.5 mL of the reservoir solution. The crystals were transferred to the cryo-protectant solution containing 30% ethylene glycol and 70% reservoir solution and flash cooled in liquid nitrogen.

Data collection was carried out at the Canadian Light Source (CLS) on beam line 08B1-1. The data sets were indexed, integrated, and scaled using XDS program (42) with AutoProcess Script (43). The structures were solved by the molecular replacement method using program Phaser in Phenix suite (44) using the structure of S-Adenosylmethionine-bound RomA (PDBID: 5CZY) as a search model. The peptides were fitted into the electron density in COOT (45) and the structures refined using Phenix software (44). The pertinent data collection and refinement information are summarized in Table 1. The diffraction data and coordinates were deposited to the Protein Databank.

### Protein purifications for HMT assays

GST fusion proteins were expressed in *E. coli* BL21-DE3 (Agilent) induced with 1 mM IPTG (isopropyl b-D-thiogalactopyranoside) and purified from clarified supernatants by affinity chromatography using glutathione agarose beads (Sigma-Aldrich #G4510) and eluted with 50 mM Tris-HCl pH9, 10 mM L-glutathione reduced (Sigma-Aldrich #G4251). The HMTase assay was carried out as described previously (8). Briefly, 500 ng of GST fusion proteins were incubated with 500 ng of recombinant histone H3 (NEB #M2503S) and 3.2 µM cold SAM (SIGMA#A007) in HMT buffer (50 mM TRIS-HCL pH8, 20 mM KCl, 250 mM sucrose 10 mM MgCl2, 1 mM DTT, protease inhibitors) for 30 min at 30°C. Reaction was stopped with Laemli buffer and samples loaded on SDS PAGE gels (Biorad). Radioactive assays were performed with 200nCi of ^14^C SAM (Perkin Elmer).

### Peptide pull down assays

Biotin-conjugated histone peptides were purchased from AnaSpec. p1 (H3_1-21aa_ ref: AS-61702): ARTKQTARKSTGGKAPRKQLA-GGK(Biotin)-NH2; p2 (H3_1-21aa_Thr6Tyr – custom peptide): ARTKQYARKSTGGKAPRKQLA-K(Biotin)-NH2; p3 (H3_1-7aa_ – custom peptide): ARTKQT-K(Biotin)-NH2. The QC data provided for purity were >95%, and the identity was confirmed by MS. One microgram of histone peptides was immobilized on Dynabeads-MyOne-Streptavidin T1 (Invitrogen#65601). Peptide-bound beads were incubated with 1 µg of GST fusion proteins in binding buffer (20 mM HEPES, pH 7.5, 150 mM NaCl, 0.25% NP-40, 1 mM EDTA, 1 mM DTT, and 20% glycerol) at 4°C overnight. Bound proteins were washed 6× with binding buffer and eluted by boiling with Laemmli buffer. Proteins were separated by SDS-PAGE followed by Western blotting with the indicated antibodies.

### Infections, immunofluorescences, and microscope analyses

THP-1 cells (ATCC TIB-202) were maintained in RPMI-1640 medium, GlutaMAX Supplemented (Gibco) at 37°C and 5% CO_2_. THP-1 were differentiated in adherent macrophages upon 72-hour incubation with phorbol 12-myristate 13-acetate PMA (50 ng/mL). Infections were carried out as previously described (8). Briefly, differentiated THP-1 cells were infected with *L. pneumophila* at a multiplicity of infection (MOI) of 10 for 6 hours. For immunofluorescence analyses, cells were fixed in 4% paraformaldehyde, permeabilized with PBS-0.1% Triton X-100, and stained with DAPI, anti-H3K14me antibody (#H3-2B10; Euromedex), and anti-LPS-Sg1 (kindly provided by Sophie Jarraud, National Reference Center for *Legionella*, Lyon,France). Immunosignals were analyzed with a Leica SP8 Microscope. Images were processed using ImageJ software.

### Western blotting

Sample proteins were prepared in Laemmli sample buffer containing 400 mM β-mercaptoethanol and loaded on SDS-PAGE gels, followed by a transfer onto a nitrocellulose membrane (0.2 µm; Trans-Blot Turbo System, Biorad). Ponceau S (Sigma) or Coomassie Brillant Blue (CBB) stainings were carried out to ensure equal protein loading. Membranes were blocked with 5% non-fat milk in TBS-Tween 0.5% for 1 hour and incubated with the respective primary antibody overnight at 4˚C. Antibodies used are anti-GST (#AB3282; Mollipore) and anti-H3K14me (#H3-2B10; Euromedex). Membranes were washed and probed with horseradish peroxidase-coupled antibody against either mouse IgG or rabbit IgG (1:2,500 in 5% non-fat milk TBS-Tween) for 1 hour (Cell Signaling Technology). The proteins were visualized by chemiluminescence detection using HRP Substrate Spray Reagent (Advansta) on the G:BOX instrument (Syngene). Images were processed and quantified using Fiji software (46).

### Real-time SPR binding studies

All experiments were performed by using a Biacore T200 instrument (Cytiva) equilibrated at 25°C. Wild-type LegAS4 and its mutated forms were immobilized on different flowcells of a Sensor Chip CM5 (Biacore AB) after the surface had been conditioned with three injections of NaOH50mM. LegAS4: 13,000 resonance units (RU ~pg/mm^2^); LegAS4-Ile447Tyr-Asn493Tyr: 14,250 RU; and LegAS4-Asn493Tyr: 11,300 RU were immobilized in flow cells 2, 3, and 4, giving three traces per injection of analyte, with flow cell 1 immobilized with GST alone and used as the reference for subtraction. H3 peptide (H3_1-21aa_ H-ARTKQTARKSTGGKAPRKQLA-OH; Anaspec #AS-61701) and SAM (SIGMA#A007) were injected in HMT buffer (50 mM Tris-HCl pH 8; 20 mM KCl; 250 mM sucrose; 10 mM MgCl2; and 1 mM DTT).

### Phylogenetic analyses

To infer the origin of the ankyrin domains of RomA, homologs of this domain were selected from representative eukaryotic organisms and from a BLASTP homology search (minimum identity threshold of 30%). The sequences were aligned using Muscle (47), and ambiguous positions were refined using BMGE (48). Tree reconstruction was performed using Likelihood through FastTree 2 (49).

## SUPPLEMENTAL MATERIAL

Figures S1 and S2 mbio.01655-23-s0001.pdf Electron density map around the peptide P1 and phylogenetic tree of the ANK domain.

ASM does not own the copyrights to Supplemental Material that may be linked to, or accessed through, an article. The authors have granted ASM a non-exclusive, world-wide license to publish the Supplemental Material files. Please contact the corresponding author directly for reuse.
