# Supplementary material for: The SET and ankyrin domains of the secreted Legionella pneumophila histone methyltransferase work together to modify host chromatin
Source: mBio. 2023 Oct 5;14(5):e01655-23. doi: 10.1128/mbio.01655-23 (PMC10653858; doi:10.1128/mbio.01655-23)
Supplement: Figures S1 and S2 — Electron density map around the peptide P1 and phylogenetic tree of the ANK domain. [file mbio.01655-23-s0001.pdf]

Supplementary Information

The SET- and Ankyrin domains of the secreted *Legionella pneumophila* histone methyltransferase work together to modify host chromatin

Monica Rolando[a], Ivy Yeuk Wah Chung [b], Caishuang Xu[b,*], Laura Gomez-Valero[a], Patrick England[c], Miroslaw Cygler[b]# and Carmen Buchrieser[a]#

[a]Institut Pasteur, Université de Paris, Biologie des Bactéries Intracellulaires, 75724, Paris, France; [b]Department of Biochemistry, Microbiology and Immunology, University of Saskatchewan, Saskatoon, Saskatchewan S7N 5E5, Canada; [c]Institut Pasteur, Université de Paris, Plateforme de Biophysique Moléculaire, CNRS UMR3528, 75724, Paris, France

Figure S1: Fo-DFc electron density map around the peptide (P1) contoured at 0.8 sigma.

Figure S2: Phylogenetic tree of the ank domain encoded by the protein RomA (Lpp1683) and selected homologs.

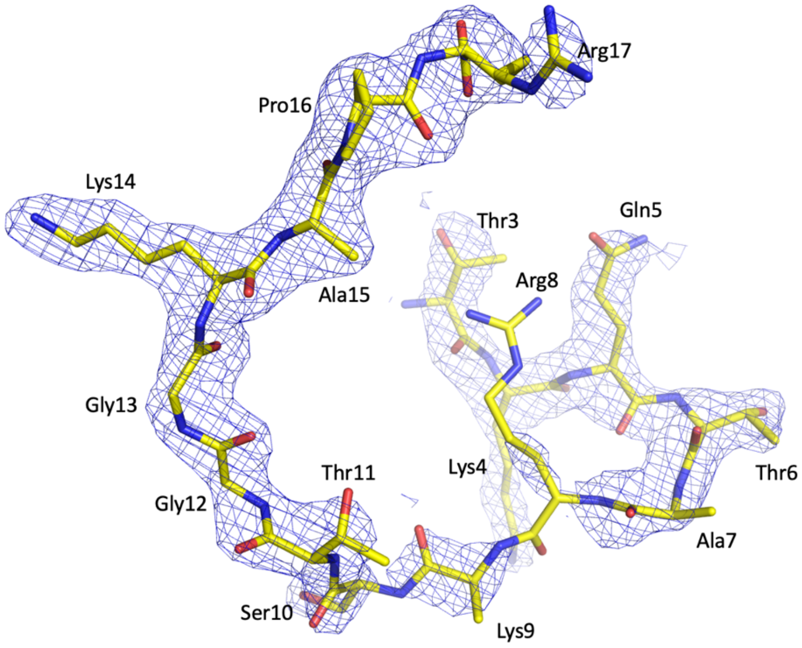

Figure S1: **Fo-DFc electron density map around the peptide (P1) contoured at 0.8 sigma.** The 2Fo-DFc electron density map contoured at 1.0 sigma level superposed on the stick representation of the peptide P1. The side chain of Lys9 is disordered while Arg8 could be traced at a lower contour level. The electron density for all other residues is well defined.

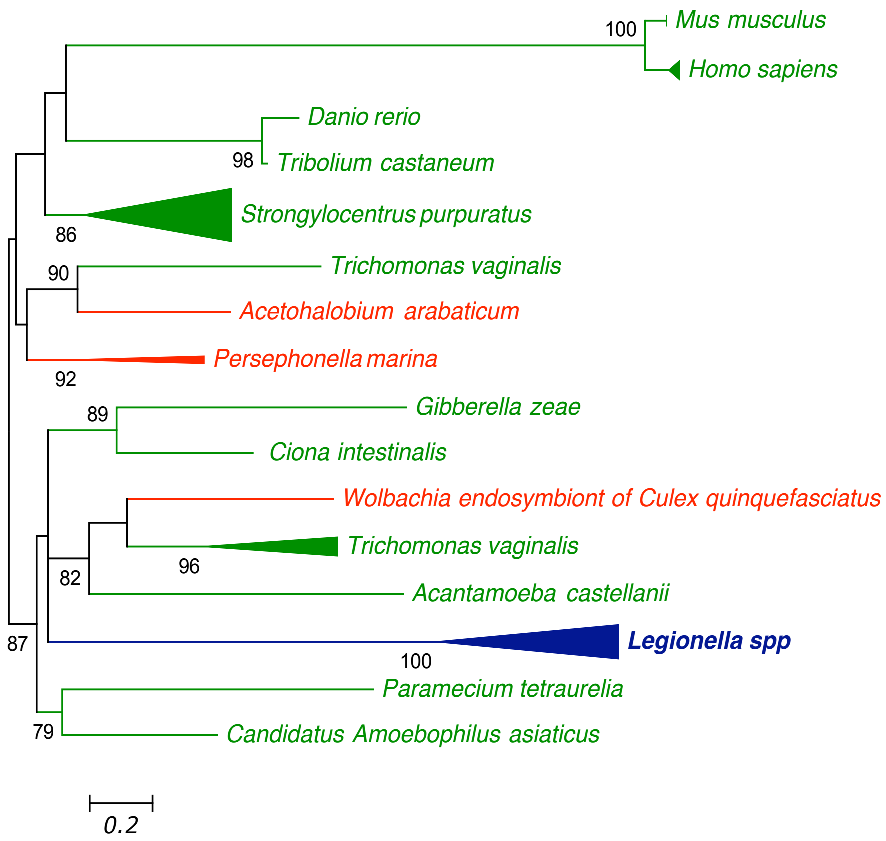

Figure S2: Phylogenetic tree of the ank domain encoded by the protein RomA (Lpp1683) and selected homologs. The Ank domain of RomA seems to have been acquired by horizontal gene transfer from a eukaryotic host. Names in green and red correspond to eukaryotic and prokaryotic organisms, respectively. Values adjacent to nodes represent local support values.
